# Paraoxonase 3 inhibits cell proliferation and serves as a prognostic predictor in hepatocellular carcinoma

**DOI:** 10.18632/oncotarget.12145

**Published:** 2016-09-20

**Authors:** Jie Cai, Sheng-Xian Yuan, Fu Yang, Qi-Fei Tao, Yuan Yang, Qing-Guo Xu, Zhen-Guang Wang, Jian Yu, Kong-Ying Lin, Zong-Yan Wang, Jin-Zhao Ma, Chuan-Chuan Zhou, Fang Wang, Shu-Han Sun, Wei-Ping Zhou

**Affiliations:** ^1^ The Third Department of Hepatic Surgery, Eastern Hepatobiliary Surgery Hospital, Second Military Medical University, Shanghai, China; ^2^ The Department of Medical Genetics, Second Military Medical University, Shanghai, China

**Keywords:** paraoxonase 3, hepatocellular carcinoma, prognostic predictor, cell proliferation, cDNA microarray

## Abstract

Paraoxonase 3 (PON3) exerts prominent anti-inflammation and anti-oxidation properties mainly at the cellular level, and is primarily expressed in the liver. However, its role in HCC remains unexplored. Here, we investigated the expression pattern, clinical significance, and function of PON3 in HCC. PON3 mRNA and protein levels were respectively determined in two large cohorts using quantitative real-time polymerase chain reaction (qRT-PCR) and immunohistochemistry (IHC) of tissue microarray. We found that PON3 was downregulated in most HCCs. Kaplan-Meier and log-rank test showed that PON3 downregulation predicted shorter recurrence-free survival (RFS) and overall survival (OS) time in all HCC patients, especially early-stage HCC patients. Cox regression analysis revealed that the PON3 downregulation was an independent risk factor for RFS and OS. Gain- and loss-of-function experiments revealed that PON3 suppressed cell proliferation *in vivo* and *in vitro*, which was attributed to its cell-cycle arrest effect. In addition, microarray analysis showed that some pro-proliferative genes were elevated when PON3 was knockdown, and these genes possibly involved in the underlying mechanisms. In conclusion, our studies reveal the cell proliferation inhibitory function of PON3 and offer a potential prognostic predictor and therapeutic target for HCC.

## INTRODUCTION

Hepatocellular carcinoma (HCC) is the fifth most prevalent tumor type and the second leading cause of cancer deaths worldwide [1]. Despite advances in diagnosis and treatment, prognosis for patients with HCC remains extremely poor [2]. Most HCCs develop in the context of severe liver fibrosis and cirrhosis, following years of chronic liver inflammation induced by viral infections, chemicals, autoimmunity, and/or metabolic diseases [3]. Currently, cancer prevention or anti-cancer therapy targeting inflammation-related molecules has been widely applied for some tumors [4, 5]. However, for HCC, a typically inflammation-related malignant tumor, inflammation-targeted therapy is still lacking. Therefore, further studies to explore the molecules link between inflammation and HCC are essential for the identification of novel monitoring and therapeutic targets for this dreadful disease.

Paraoxonase (PON) proteins have been reported to markedly prevent oxidative stress and inhibit inflammation [6]. The PON gene family consists of three members, PON1, PON2, and PON3, located adjacent to each other on chromosome 7q21.3-q22.1 in humans and shares high levels of homology in amino acid and nucleotide sequences [7]. The expression and specific activities of PON genes were found to negatively correlate with several inflammatory disorders, such as cardiovascular diseases, type-2 diabetes, and inflammatory bowel disease [8]. However, their roles in cancer are rarely evaluated, and despite being mainly expressed in the liver, the effect of PONs in the development and progression of HCC has not been explored.

PON3 is the most recently identified and least studied among the three PON genes. Unlike the ubiquitously expression of PON2, PON3 is primarily expressed in the liver, and to a much lesser extent in the kidney [9]. PON1 is also synthesized in the liver and is secreted into the serum where it performs antioxidant and anti-inflammatory activities [10]. However, the similar protective activities of PON3 are thought to take place mainly at the cellular level, namely in hepatocytes [11, 12]. Moreover, rabbit serum PON3 has a significantly more pronounced antioxidant effect when compared with serum PON1 [13], and serum PON3 concentration is also associated with the severity of hepatic impairment in patients with chronic liver disease [14]. All these reported findings suggest that PON3 is more closely related to HCC progression than PON1 and PON2. Therefore, in this study, we focused on the expression pattern, clinical significance, and function of PON3 in HCC.

## RESULTS

### PON3 expression is frequently decreased in human HCC tissues

**Figure 1:**
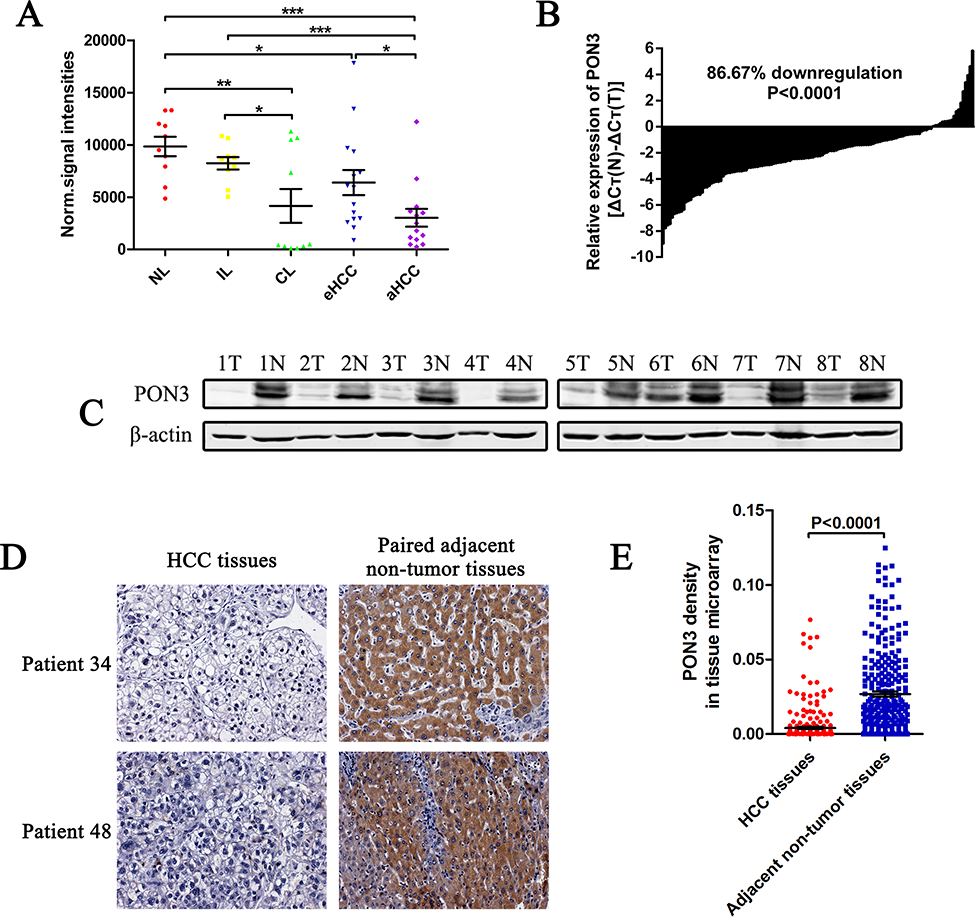
PON3 is frequently decreased in HCC (**A**) Normalized signal intensities of PON3 in normal liver (NL), chronic inflammatory liver (IL), cirrhotic liver (CL), early-stage HCC (eHCC), and advanced-stage (aHCC) tissues in a microarray (GSE54238). (**B**) PON3 mRNA level in 135 paired HCC and the adjacent non-tumor tissues were evaluated by qRT-PCR. (**C**) Western-blots showing PON3 protein level in tumor tissues (T) and the paired adjacent non-tumor tissues (N) from eight HCC patients. (**D**) Two representative cases of PON3 IHC staining in HCC and adjacent non-tumor tissue pairs in tissue microarray. (**E**) Relative IHC staining of PON3 in paired HCC and adjacent non-tumor tissue samples (*n* = 286). Statistical significance was determined by student's *t*-tests. *p < 0.05; **p < 0.01; ***p < 0.0001 compared with respective controls. Data are shown as mean ± SD.

Schweikertet al. reported PON3 was upregulated in several types of cancer tissues [15], which appear to contradict to its protective role observed in other inflammatory disorders. To determine PON3 expression pattern in HCC, we first analyzed a public GEO DataSets (GSE14520). The results showed that PON3 and PON1 were significantly downregulated in HCC (Figure S1A, S1B), whereas PON2 was upregulated (Figure S1C). Further analyses of our own previously constructed microarray data (GSE54238) showed that PON3 was progressively downregulated as normal liver develops into chronic hepatitis, cirrhosis, early-stage HCC, and advanced HCC (Figure 1A).

To clarify the significance of PON3 expression in HCC, PON3 mRNA and protein levels in fresh frozen HCC and the adjacent non-tumor tissues were evaluated. In the qRT-PCR cohort including 135 pairs of HCC and the adjacent non-tumor tissues, PON3 was markedly downregulated in HCC tissues of most patients (86.67%) (Figure 1B). Western-blot results showed that PON3 protein level was also downregulated in HCC when compared with the adjacent non-tumor liver tissues (Figure 1C). Furthermore, we constructed a tissue microarray containing 286 pairs of HCC and the adjacent non-tumor tissues for IHC staining. PON3 staining was enriched in the cytoplasm as previously reported [15], and PON3 staining was significantly reduced in HCC tissues (Figure 1D). Methodology to quantitate the staining level has been described [16]. In agreement with the qRT-PCR and Western-blot results, the protein level of PON3 was significantly lower in most HCC tissues (253 of 286 patients) in the tissue microarray cohort (Figure 1E). In contrast to a previous finding [15], our results revealed that PON3 was downregulated in HCC, implying its anti-oncogenic role in HCC.

### PON3 downregulation is negatively associated with malignant clinicopathological characteristics and predicts poorer prognosis in HCC patients following hepatectomy

Clinical role of PON3 in tumor is yet to be reported. The tissue microarray cohort (286 patients) was further divided into the low and high subgroups according to the median PON3 density in the HCC tissues. Subsequently, several standard clinicopathological features were collected and analyzed. Noticeably, we found strong negative correlations between PON3 expression and many progressive clinical features, including poor differentiation (*p* < 0.001), tumor size ≥ 5 cm (*p* = 0.008), absence of encapsulation (*p* = 0.026), serum AFP levels of ≥ 20 μg/L (*p* < 0.001), and early recurrence (*p* = 0.010) (Table 1).

**Table 1:** Correlation of PON3 protein level in HCC tissues with clinicopathological characteristics

Variable	PON3 expression^▲^		χ^2^	*p*-value
	Low (*n* = 143)	High (*n*= 143)		
**Age (year), < 50: ≥ 50**	80:63	68:75	2.016	0.156
**Gender, Male: Female**	130:13	124:19	1.267	0.260
**Hepatocirrhosis, Absent: Present**	96: 47	102: 41	0.591	0.442
**Edmondson grade, I + II: III + IV**	14:129	53:90	29.647	0.000^★^
**Tumor size (cm), < 5: ≥ 5**	85:58	106:37	6.951	0.008^★^
**Capsule, Absent: Present**	49:94	32:111	4.978	0.026^★^
**Microvascular invasion, Absent: Present**	130:13	136:7	1.935	0.164
**Satellite lesions, Absent: Present**	133:10	131:12	0.197	0.657
**TNM stage, I:II–IV**	114:29	111:32	0.188	0.665
**HBeAg, Negative: Positive**	92:51	91:52	0.015	0.902
**Serum AFP (ug/L), < 20: ≥ 20**	46:97	80:63	16.400	0.000^★^
**Early recurrence, Absent: Present**	78:65	100:43	7.201	0.010^★^

Data are expressed as ratios.

▲ The patients were divided into the low and high subgroups according to the median PON3 density in the HCC tissues.

★ *p* < 0.05 by χ test.

**Figure 2:**
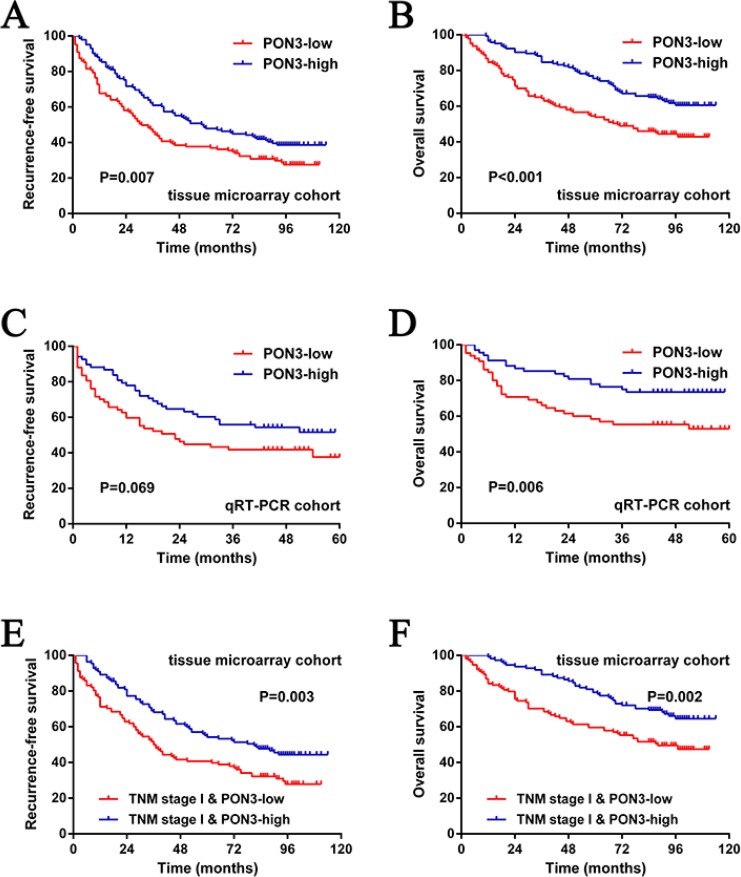
PON3 can serve as a prognostic predictor in patients with HCC (**A** and **B**) The low PON3 subgroup had significantly shorter RFS and OS than the high PON3 subgroup in the tissue microarray cohort. (**C** and **D**) Similar results were observed in the qRT-PCR cohort. (**E** and **F**) The prognostic value of PON3 was also observed in patients with early-stage HCC (TNM stage I). Statistical significance was assessed by two-sided log-rank tests.

To determine the prognostic value of PON3 in HCC, Kaplan-Meier survival curves were generated and log-rank tests were performed in both tissue microarray and qRT-PCR cohorts. The median expression level was used as the cutoff. Remarkably, we found that patients with lower PON3 protein level had significantly shorter RFS (median RFS times of 28 and 52 months, for the low and high PON3 subgroup, respectively; *p* = 0.007) and OS (median OS times of 68 and 92 months, for the low and high PON3 subgroup, respectively; *p* < 0.001) than patients with higher PON3 protein level (Figure 2A, 2B). Moreover, similar findings were found in the qRT-PCR cohort although there was no statistically significant difference for RFS, which can be due to the small sample size or short follow-up period (*p* = 0.069 for RFS, *p* = 0.006 for OS) (Figure 2C, 2D). An additional GEO DataSets (GSE14520) analyses supported the prognosis-predictive value of PON3 mRNA level in HCC (Figure S2A, S2B).

Univariate analysis performed with the tissue microarray cohort revealed that hepatocirrhosis, satellite lesions, TNM stage, serum AFP, and PON3 expression level could serve as predictors for RFS. Similarly, gender, Edmondson grade, tumor size, TNM stage, serum AFP, and PON3 expression level could be predictor for OS (Table S1). These statistically significant clinicopathological parameters were enrolled for multiple analyses. A Cox's proportional hazards regression analysis indicated that PON3 downregulation was an independent risk factor for both RFS (hazard ratio [HR] of 1.379, 95% confidence interval [CI] of 1.021-1.863, *p* = 0.036) and OS (HR of 1.687, 95% CI of 1.173-2.426, *p* = 0.005) in patients with HCC after curative hepatectomy (Table 2).

**Table 2:** Multivariate analysis of the risk factors for OS and RFS

Variable^▲^	Recurrence-Free Survival		Overall Survival	
	Hazard ratio (95% CI)	*p*-value	Hazard ratio (95% CI)	*p*-value
**Gender, Female**	--	--	0.446 (0.225-0.885)	0.021^★^
**Hepatocirrhosis, Present**	1.414 (1.040-1.922)	0.027^★^	--	--
**Edmondson grade, III + IV**	--	--	0.845 (0.519-1.377)	0.500
**Tumor size (cm), ≥ 5**	--	--	1.671 (1.166-2.395)	0.005^★^
**Satellite lesions, Present**	1.478 (0.865-2.525)	0.153	--	--
**TNM stage, II−IV**	1.644 (1.145-2.361)	0.007^★^	1.934 (1.327-2.819)	0.001^★^
**Serum AFP (ug/L), ≥ 20**	1.274 (0.941-1.726)	0.117	1.689 (1.152-2.476)	0.007^★^
**PON3 expression, Low**	1.379 (1.021-1.863)	0.036^★^	1.687 (1.173-2.426)	0.005^★^

▲ Variables were adopted for their prognostic significance by univariate analysis.

★ *p* < 0.05 by Cox proportional hazards regression model.

TNM staging system has been used to predict outcomes of HCC patients, but association between the TNM stage and the actual outcome is not always observed. The prognosis of some patients with early-stage HCC still turn out to be poor, suggesting that a supplementary prognostic predictor is required for these patients. Therefore, patients with early-stage HCC (TNM stage I) were stratified and subgroup analyses were performed. Notably, the prognosis-predictive value of PON3 in early-stage HCC (TNM stage I) was still proven (*p* = 0.005 for RFS, *p* = 0.002 for OS) (Figure 2E, 2F). Similar results were also observed in patients with normal serum AFP level (< 20 μg/L), poor tumor differentiation (Edmondson grade III + IV), and absence of hepatocirrhosis (Figure S2C–S2H).

Collectively, we demonstrated that PON3 can be used as a prognostic predictor for HCC patients after hepatectomy, especially those with early-stage HCC (TNM stage I).

### PON3 suppresses HCC tumor growth *in vivo*

As described above, PON3 downregulation was found in HCC in the present study and its expression was negatively associated with several progressive clinicopathological features, including tumor size. To elucidate the pro-tumorigenic or anti-tumorigenic properties of PON3 in HCC, a mouse xenograft tumor model was utilized. Before the essays were performed, expression level of PON3 in several HCC cell lines was examined (Figure S3A). SMMC-7721, HCC-LM3, Huh7, and HepG2 cells were selected to generate stable cell lines with PON3 over-expression or knockdown (which we named SMMC-7721-PON3, HCC-LM3-PON3, Huh7-SH1 or -SH2, and HepG2-SH1 or -SH2), using recombinant lentiviruses containing full-length PON3 or shRNAs targeting PON3. The efficiency of PON3 overexpression or knockdown was confirmed by qRT-PCR and Western-blot (Figure S3B, S3C).

**Figure 3:**
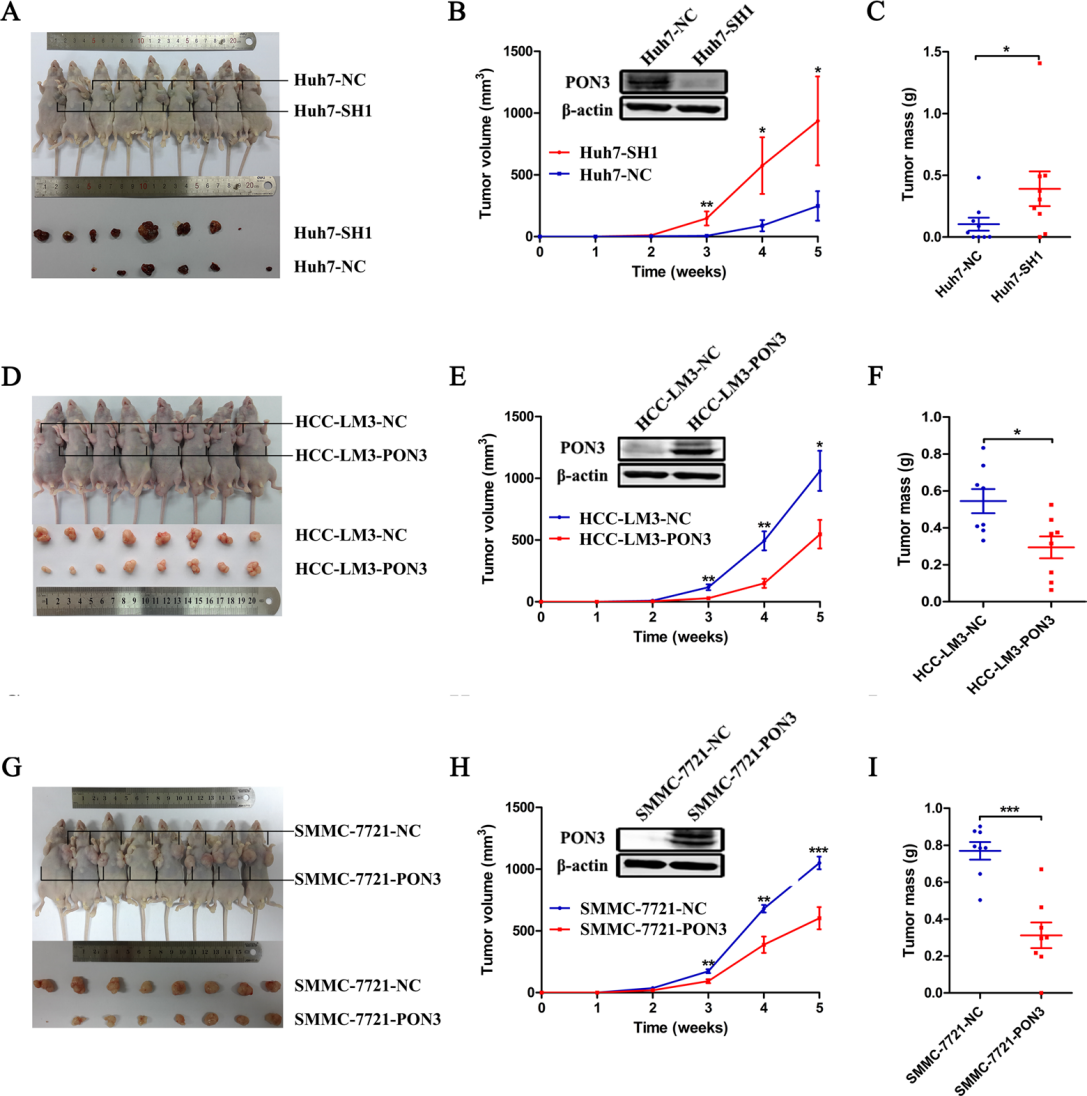
PON3 suppresses tumor growth *in vivo* A mouse xenograft tumor model was utilized. Mice received subcutaneously injections of 1 × 10^7^ (**A**–**C**) Huh7-SH1 knockdown and Huh7-NC control cells, (**D**–**F**) HCC-LM3-PON3 overexpression and HCC-LM3-NC control cells, or (**G**–**I**) SMMC-7721-PON3 overexpression and SMMC-7721-NC control cells. (A, D, and G) Mice and tumors were evaluated five weeks post-injections and representative images are shown. (B, E, and H) Tumor growth curve and (C, F, and I) tumor weight during the five weeks study period are shown. Data are shown as mean ± SEM. Statistically significant differences were determined by student's *t*-tests. *p < 0.05; **p < 0.01; ***p < 0.0001 compared with the respective controls.

Equal numbers (1 × 10^7^) of Huh7-SH1 cells and its control Huh7-NC cells were subcutaneously injected into the left- and right-armpit of each nude mouse, respectively. Our results showed that knockdown of PON3 evidently promoted tumor growth. A significant difference in tumor volume was observed three weeks post-injection (*p* = 0.008), and was increased throughout the study period until the experimental endpoint, at which the average tumor weight was approximately four-fold higher in tumors with PON3 knockdown (W = 937 mg) when compared with control tumors (W = 248 mg) (Figure 3A–3C). In the same way, xenograft tumor formation in nude mice was also assessed using cells with exogenous PON3 overexpression and their respective controls. As shown in Figure 3D–3F, tumors with PON3 overexpression (HCC-LM3-PON3) showed a significantly decreased tumor growth compared with its control (HCC-LM3-NC), reflected by tumors' volume and weight (*p* = 0.046 for tumor volume, *p* = 0.021 for tumor weight). Parallel results were also obtained using another stable PON3 overexpression cell lines (Figure 3G–3I). These results revealed that PON3 was capable of suppressing tumor growth *in vivo* and acted as an anti-oncogene in HCC, indicating that PON3 is a potential target for HCC molecular therapy.

### PON3 inhibits cell proliferation and clonogenicity of HCC cells *in vitro*

**Figure 4:**
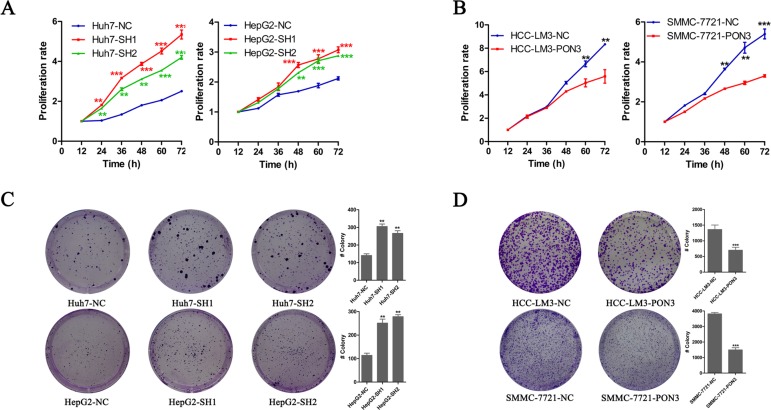
PON3 inhibits cell proliferation and clonogenicity in HCC cells (**A** and **B**) Cell proliferation was determined by CCK-8 assay, and growth curves were generated by reading the absorbance value at different time points. Growth curves of (A) PON3 knockdown and (B) PON3 overexpression cells and their respective controls are shown. (**C** and **D**) Representative results of colony formation assays of (C) PON3 knockdown cells, (D) PON3 overexpression cells and their respective controls. *p < 0.05; **p < 0.01; ***p < 0.0001 compared with the respective controls. Significance was determined from three independent experiments and assessed by student's t-test. Data are shown as mean ± SD.

Here, CCK8 was utilized to assess HCC cells proliferation *in vitro*. As shown in Figure 4A, PON3 knockdown promoted a remarkable cell proliferation in Huh7 and HepG2 cells, and differences in cell growth between the knockdown and control cells continued to expand in subsequent time points. Conversely, HCC-LM3 and SMMC-7721 cells with PON3 over-expression showed notable reductions in proliferation rate compared with their respective control cells (Figure 4B). To evaluate the long-term effect of PON3 on cell proliferation, colony-formation assays were performed. As shown in Figure 4C and 4D, number of tumor colonies formed at day 14 was much more in PON3 knockdown cells and markedly reduced in PON3 overexpression cells. These findings were consistent with our *in vivo* tumor growth results and further confirmed the inhibitory effect of PON3 in cell proliferation.

### The inhibitory function of PON3 in cell proliferation is attributed to cell-cycle arrest

To further explore whether PON3 suppressed HCC cells proliferation by regulating apoptosis or cell cycle, a series of assays were performed. In the same study, Schweikertet al. reported that PON3 acted as an oncogene by inhibiting cell apoptosis [15]. In contrast, our findings suggested the tumor-suppressive function of PON3 in HCC. Therefore, we examined the anti-apoptotic effect of PON3 in HCC cells using flow cytometric analyses. Surprisingly, no significant difference in apoptosis was found, either in PON3 overexpression or knockdown stable cell lines when compared with their respective controls, following treatment with apoptosis-inducers A (Apopida) and B (Apobid) for 6 h (Figure S4A). Levels of apoptosis-associated proteins in Huh7-SH1, -SH2, HCC-LM3-PON3, and their respective controls were also evaluated by Western-blot. Similarly, no difference was observed in apoptosis-associated protein levels (Figure S4B). TUNEL staining assays further confirmed our findings (Figure S4C).

**Figure 5:**
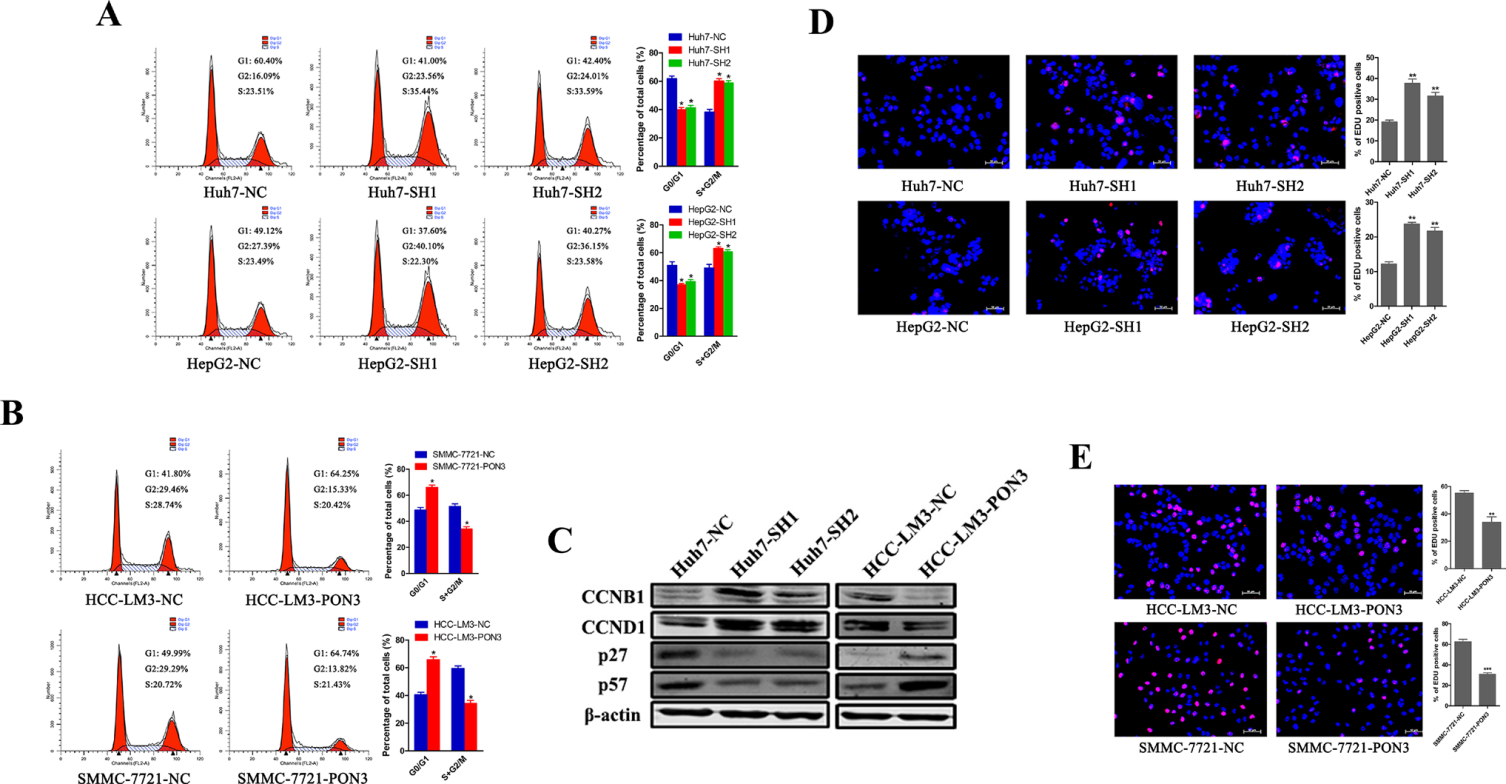
PON3 induces cell cycle arrest in HCC cells A flow cytometric analysis of cell cycle phase distribution of (**A**) PON3 knockdown cells and (**B**) PON3 overexpression cells. (**C**) Cells with PON3 knockdown and overexpression were collected for western-blot analysis of cell-cycle regulatory proteins (CCNB1, CCND1, p27, p57). EdU immunofluorescence assays were performed in (**D**) PON3 knockdown cells and (**E**) PON3 overexpression cells. EdU-positive cells were stained red. Magnification, 200×; scale bars = 50 μm. Significance was assessed by student's *t*-test. *p < 0.05; **p < 0.01; ***p < 0.0001 compared with the respective controls. The experiments were performed in triplicate and the data are shown as the mean ± SD.

However, flow cytometric analyses of the cell-cycle revealed a significant reduction of cells in the G1 phase and a significant increase of cells in the S and G2 phase, upon PON3 knockdown compared with the control cells (Figure 5A). Furthermore, over-expression of PON3 blocked G1/S transition and induced cell cycle arrest, reflected by an increase in percentage of HCC cells in G1 phase and a decrease in the percentage of cells in the S and G2 phase (Figure 5B). Cell-cycle regulatory proteins detected by Western-blot also supported our findings. The results showed that the CCNB1 and CCND1 proteins were increased with the knockdown of PON3 and decreased with the overexpression of PON3, p27 and p57 proteins presented reversed results (Figure 5C). EdU assays further confirmed the results that the amount of proliferating cells was increased upon PON3 knockdown, and decreased upon PON3 overexpression (Figure 5D and 5E).

Together, these results revealed that the cell proliferation-suppressive function of PON3 in HCC could be attributed to its cell cycle arrest property, and not due to its protective apoptotic function.

### A cluster of pro-proliferative genes are elevated when PON3 is knocked down

**Figure 6:**
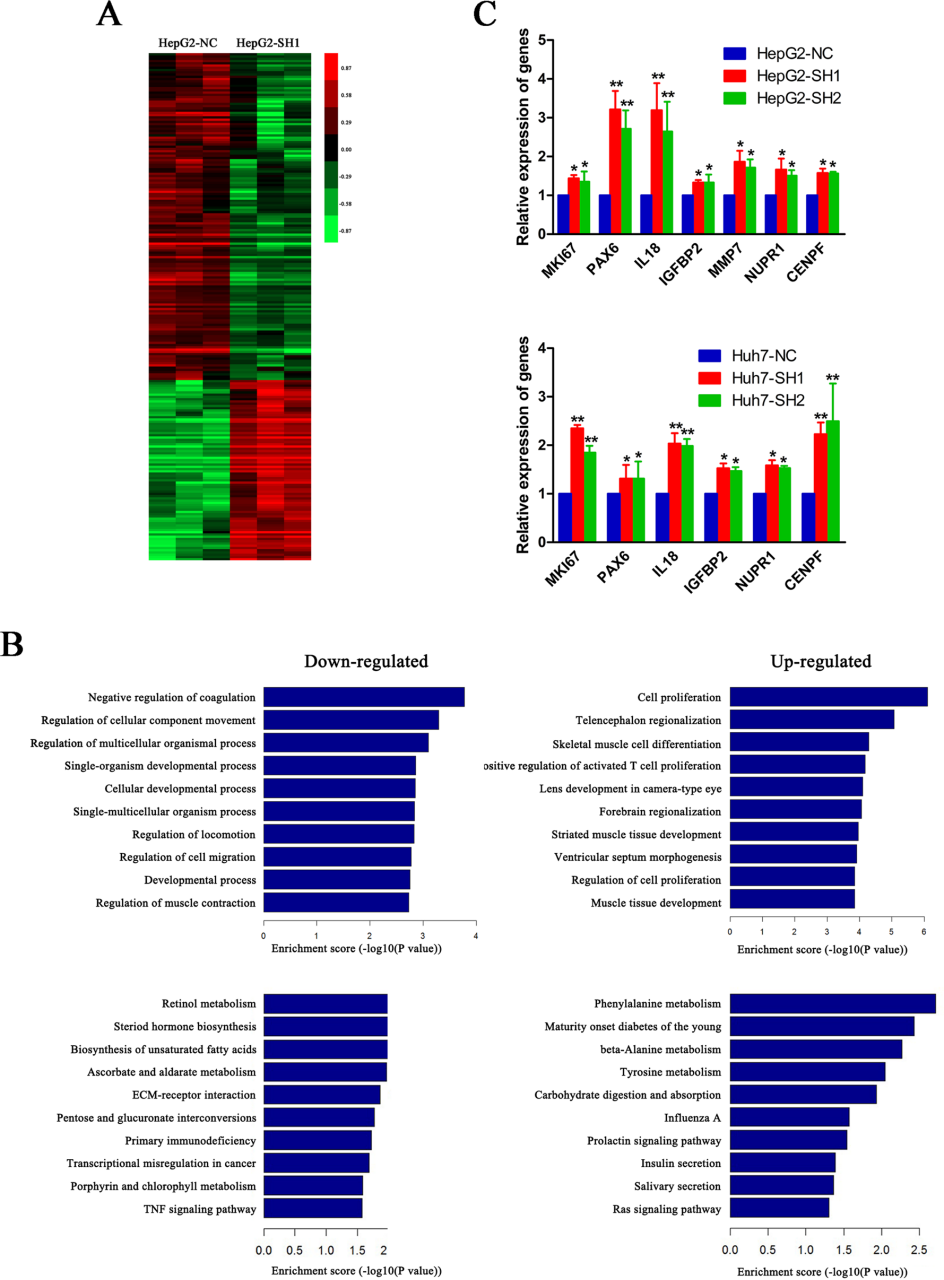
A cluster of pro-proliferative genes are elevated when PON3 is knocked down (**A**) A heatmap of differentially expressed genes in HepG2-SH1 cells compared with the control cells. Up- and down-regulated genes are represented with red and green colors, respectively. (**B**) A GO biological process analysis and pathway enrichment analysis of down-and up-regulated genes in HepG2-SH1 cells compared with the control cells. (**C**) Relative expressions of MKI67, PAX6, IL18, IGFBP2, MMP7, NUPR1, and CENPF in HepG2 and Huh7 cells with stable PON3 knockdown and their respective control cells were validated by qRT-PCR. *p < 0.05; **p < 0.01 compared with the respective controls. The experiments were repeated more than three times. Data are expressed as the mean ± SD and compared by student's *t*-test.

To investigate the potential genes involved in the suppression of PON3 in cell proliferation, cDNA microarrays were constructed. The gene expression data was obtained from three independent competitive hybridizations, comparing PON3 knockdown HepG2 cells (HepG2-SH1) and control cells (HepG2-NC). The criteria of corrected *p*-value < 0.05 and absolute fold change > 1.5 were used to identify differentially expressed genes. A total of 225 genes were found to be differentially expressed, 145 genes were downregulated, and 80 genes were upregulated. These differentially expressed genes were used to generate a heatmap. As shown in Figure 6A, they were clearly segregated into HepG2-NC and HepG2-SH1.

Subsequently, these up- and down-regulated genes were separately subjected to GO and pathway enrichment analyses (Figure 6B). In the upregulated genes subgroup, we found “cell proliferation” was the most enriched GO biological process (*p* = 7.77 × 10^−7^), containing MKI67, PAX6, IL18, IGFBP2, MMP7, NUPR1, CENPF, and others, which were known to play positive roles in cell proliferation [17–23]. Additionally, seven of the top ten categories in the upregulated genes subgroup were associated with development, differentiation, and proliferation, which further suggested the vital role of PON3 in cell proliferation. Meanwhile, in both down- and up-regulated genes, a pathway enrichment analysis (KEGG pathways) revealed disruption of many metabolism pathways. The retinol metabolism, the steroid hormone biosynthesis, the biosynthesis of unsaturated fatty acids, and the ascorbate and aldarate metabolism were disrupted in the downregulated genes group, while the phenylalanine metabolism, the beta-alanine metabolism, and the tyrosine metabolism were disrupted in the upregulated genes group. These factors which may contribute to the cell proliferation increase when PON3 was knocked down.

To validate the gene expression profiling results, the seven aforementioned differentially expressed genes were selected for qRT-PCR analyses. In agreement with the microarray results, expression of these pro-proliferative genes were induced when PON3 was knocked down in HepG2 and Huh7 cells (MMP7 expression was below the limit of detection in Huh7) (Figure 6C). These altered expressions of proliferation-related genes may participate in the proliferation-inhibitory effect of PON3 in HCC cells.

## DISCUSSION

Prolonged inflammation may trigger various potentially damaging processes, such as the induction of DNA damage through reactive oxygen species (ROS) accumulation, leading to cancer initiation and progression [24, 25]. PON3 is mainly expressed in the liver, and exerts anti-inflammatory and anti-oxidative properties in cellular lever, namely in hepatocytes [9, 11, 12]. PON3 was reported as a more efficient inhibitor of oxidative stress than PON1 in rabbit serum [13]. In addition, serum PON3 concentration was associated with the severity of hepatic impairment in patients with chronic liver disease [14], and PON3 knockout mice exhibited altered bile composition along with a dramatic increased plasma levels of alanine aminotransferase, aspartate aminotransferase, and direct bilirubin [26]. For PON1, Akkiz et al. reported that neither the Q192R polymorphism nor the L55M polymorphism had relationship with the risk of developing HCC [27]. All these findings suggest that PON3 is more hepatoprotective, and may suppress the progression of liver cancer. Therefore, we focused on the expression pattern, clinical significance, and function of PON3 in HCC here.

GEO DataSets analyses (GSE14520, GSE54238) and clinical tissues detections all revealed the downregulation of PON3 in HCC, which contradicted the results of the previous study [15], indicating that other functions of PON3 may exist in HCC. Prognostic analyses showed that patients with lower PON3 expression levels had shorter RFS and OS. A multivariate analysis further revealed that the PON3 expression level was an independent risk factor for both RFS and OS.

A subset of patients with early-stage HCC, who are predicted to have better outcomes using the standard staging system, still show poor prognosis instead, suggesting that a complementary prognostic predictor is needed for these patients. Here, further prognostic analyses revealed that early-stage HCCs with lower PON3 expression also had poorer RFS and OS (*p* = 0.003 for RFS, *p* = 0.002 for OS). These results indicated that PON3 protein measurement maybe helpful for clinicians to identify early-stage patients with high recurrence risk, and to recommend closer follow-up and appropriate adjuvant therapies for these patients.

The negative relation of PON3 and tumor size had been proved above, implying that PON3 may inhibit HCC cells proliferation. Subsequently, we found that silencing PON3 by specific shRNA accelerated tumor growth *in vivo*, whereas PON3 over-expression led to a remarkable suppression of tumor growth. Similar results were also observed using CCK8 and colony-formation assays *in vitro*. We also demonstrated that PON3 did not inhibit apoptosis in HCC cells, but induced cell-cycle arrest, which leaded to the inhibitory effect of PON3 in cell proliferation. These results also suggested PON3 as a potential therapeutic target for HCC. To further explore the potential molecular mechanism, gene expression profiles of PON3 knockdown cells were assessed. We identified 225 differentially expressed genes. Of these, a number of pro-proliferative genes, including MKI67, PAX6, IL18, IGFBP2, MMP7, NUPR1, and CENPF were found to be upregulated. These genes were previously reported to stimulate cell proliferation or cell cycle, and some of them were found to be upregulated in several solid tumors [17–23]. To the best of our knowledge, our study is the first to report a genome-wide gene expression modulation following PON3 knockdown. These results may provide clues on the detailed molecular basis of PON3-mediated suppression of HCC growth.

Little is known on the regulatory pathway of PON3. Unlike PON1 and PON2, which were reported to be down- and up-regulated, respectively, in response to oxidative stress [28], PON3 expression is not regulated by oxidative stress [9]. Moreover, several proinflammatory cytokines including IL1, IL6, and TNFα were reported to regulate PON1 [29]. Rothem et al. reported that PON3 was infrequently detected in colonic biopsy samples of patients with active ulcerative colitis or Crohn's disease compared with healthy individuals, indicating that inflammation and oxidative stress may downregulate PON3 expression in the intestine of patients with inflammatory bowel disease [30]. However, analogous results were not observed in HCC cells. PON3 expression was not significantly changed when cells were co-cultured with IL-1β, IL6, IL8, or TNFα at increasing concentrations (10, 25, 50, 100 μg/mL) or incubation time (6, 12, 24, 48 h) (data not shown). Several scenarios may explain this contradiction: 1) First, inflammation indeed did not regulate PON3 expression in HCC cells; 2) the concentration and duration of cytokine stimulation were not appropriate for HCC cells; and 3) the enrolled four cytokines were not in the range of cytokines which regulate PON3 expression. Therefore, further studies are needed to elucidate PON3 regulation, including regulation by transcription factors and epigenetic modification.

However, we were able to validate the negative association between PON3 expression and the expression of many proinflammatory factors in HCC by analyzing public data available on the GEO database (data not shown). Given the fact that proinflammatory factors did not regulate PON3 expression in HCC, these results implied that inflammation disorder might be a consequence, but not an inducer of PON3 downregulation. This was partly supported by the reduced inflammatory response observed in a PON3 overexpression study [31]. In contrast to other solid organs, liver has a prominent unique ability to regenerate following toxic injury, chronic inflammation, and surgical resection [32]. Sustained hepatic inflammation results in ROS accumulation and then triggers compensatory proliferation, which can lead to cirrhosis and further development of HCC [33, 34]. Downregulation of PON3, which leads to an exacerbated inflammatory response, may be one of the initiation factors in these progressions.

In conclusion, we investigated the expression of PON3 in HCC and its clinical value as a prognostic predictor for patients with HCC, especially at early-stage HCC. We also demonstrated the proliferation-inhibitory property of PON3 in HCC cells, suggesting PON3 as a potential therapeutic target for HCC. A microarray analysis revealed that a cluster of pro-proliferative genes were elevated when PON3 was downregulated, which may shed light on the underlying mechanisms.

## MATERIALS AND METHODS

### Patient characteristics and tissue specimens

All patients in our study were with HBV infection. We obtained 135 paired samples of HCC and the adjacent non-tumor tissues for qRT-PCR analysis. A tissue microarray containing 286 pairs of HCC and the adjacent non-tumor tissues was constructed for IHC test. HCC differentiation was defined according to the Edmondson-Steiner classification. Micrometastases were defined as tumors adjacent to the border of the primary tumor, which could only be observed under a microscope. Tumor staging was defined according to the TNM staging system. All tissue samples were randomly collected at the Eastern Hepatobiliary Surgery Hospital (Shanghai, China) from September 2005 to March 2012 and were stored at −80°C until further use. Two pathologists re-evaluated the tissues independently. The study was approved by the ethics committee of the Eastern Hepatobiliary Surgery Hospital, and written informed consents were obtained from all study participants according to the policies of the committee. RFS was calculated from the date of tumor resection until the detection of tumor recurrence, death from a non-HCC cause, or last follow-up visit. OS was defined as the length of time between surgery and either the death of the patient or the last follow-up visit. Information that could reveal the identity of the patients was excluded from this report.

### Cell culture

Hepatocellular carcinoma cell lines (SMMC-7721, HCC-LM3, Huh7, HepG2) were obtained from the China Center for Type Culture Collection (Wuhan, China) and were authenticated by the provider using DNA-fingerprinting or isoenzyme analysis. All cell lines were maintained in DMEM medium (HyClone, UT, USA) containing 10% fetal bovine serum and 1% penicillin/streptomycin (Gibco BRL, MD, USA) at 37°C, in a humidified atmosphere of 5% CO_2_.

### RNA extraction, preparation of cDNA, and qRT-PCR analysis

Total RNA was extracted from frozen tissues or cell lines using the Trizol reagent (Takara, Dalian, China) according to the manufacturer's instruction. For reverse transcriptions, 1–2 μg of total RNA, random primers, and the M-MLV Reverse Transcriptase Kit (Invitrogen, CA, USA) were used. Real-time PCR was performed using the SYBR Green Master Mix (Takara, China) in a StepOne Plus system (Applied Biosystems, CA, USA) with β-actin as an endogenous control. The qRT-PCR primers are listed in Table S2. The relative expression of RNAs was calculated using the comparative Ct method.

### Western-blot analysis

Western-blot was performed as described previously [35]. Antibody dilutions were 1:1,000 for the PON3 polyclonal antibody (Abcam, MA, USA) and 1:5,000 for β-actin (Sigma-Aldrich, USA). Apoptosis antibodies were purchased from Cell Signaling Technology (#9915; MA, USA) and diluted at 1:1,000. Cell cycle antibodies (CCNB1, CCND1, p27, p57) were purchased from AbSci (MD, USA) and diluted as the manufacturer's instruction.

### Tissue microarray, immunohistochemistry and immunohistochemical quantification

Tissue microarray (TMA) containing 286 pairs of HCC and the adjacent non-tumor tissues was constructed in collaboration with the Shanghai Outdo Biotech Company (Shanghai, China). Immunohistochemistry was performed as previously described [16]. A PON3 polyclonal antibody (Abcam, MA, USA) diluted at 1:300 was used as the primary antibody.

Density of PON3-positive staining was quantified using a DFC420 CCD camera connected to a DM IRE2 microscope (Leica Microsystems Imaging Solutions Ltd, Cambridge, United Kingdom). Photographs of representative fields were captured under a high-power magnification (200×) using the Leica QWin Plus v3 software. The Image-Pro Plus v6.0 software (Media Cybernetics Inc, MD, USA) was used to count the integrated optical density (IOD) of each photograph as previously described [16], and the ratio of IOD to total tissue area (AREA) of each photograph was calculated as PON3 density.

### Generation of stable cell lines with PON3 overexpression or knockdown

Recombinant lentiviruses containing full-length PON3 (LV-PON3), PON3 control (LV-PON3-NC), shRNAs targeting PON3 (LV-shRNA1 and LV-shRNA2), and control shRNA (LV-shRNA-NC) were purchased from Obio Technology Co., Ltd. (Shanghai, China). The shRNA target sequences were as follow: shRNA1 (F: 5′-CAGUGGUGGAUUUGACAAATT-3′, R: 5′-UUUGUCAAAUCCACCACUGTT-3′), and shRNA2 (F: 5′-CCAGAAGUAUGUCUAUGUATT-3′, R: 5′-UACA UAGACAUACUUCUGGTT-3′). Suitable concentration of puromycin was used to select for stable cells.

### Animal studies

Animal studies were approved by the Institutional Animal Care and Use Committee of the Second Military Medical University, Shanghai, China. Male athymic BALB/c nude mice (4–5 weeks old) were used and received humane care throughout the experiments. Equal number (1 × 10^7^) of transduced Huh7, HCC-LM3, or SMMC-7721 cell lines and the respective control cells were injected subcutaneously into the bilateral armpit of each mouse. Tumor length (L) and width (W) was measured weekly after injection. Tumor growth curves were plotted using the tumor volume (V = 0.5 × L × W^2^) at each time point. All mice were sacrificed five weeks following injection.

### Cell Counting Kit-8 (CCK8) assay and colony-formation assay

Approximately 3 × 10^3^ HCC cells were plated in 96-well plates, and the viability of the cells was assessed from 3 replicates in 3 independent experiments by the CCK8 (Dojindo, Kumamoto, Japan) every 12 hours. Cell proliferation curves were plotted using absorbance values at each time point.

For colony-formation, cells were seeded onto 6-well plates at a density of 1.5 × 10^3^ cells per well. After 2 weeks of growth, surviving colonies were fixed with 4% paraformaldehyde, stained with 0.1% crystal violet, and counted.

### Apoptosis analyses

Cells were pretreated with apoptosis-inducers A (Apopida) and B (Apobid) (1: 1,000, Beyotime, China) for 6 h [36]. Subsequently, cells were trypsinized, rinsed with PBS, resuspended in 1× binding buffer, stained with annexin V-FITC/PI (BD Bioscience, CA, USA), and analyzed with a flow cytometer (BD Biosciences, CA, USA).

Apoptotic cells were also identified using the One Step TUNEL Apoptosis Assay Kit (Beyotime, China) according to the manufacturer's instruction. Images were captured after counterstaining with Hoechst 33342 (Beyotime, China). The percentage of TUNEL-positive cells was evaluated and shown.

### Cell cycle analysis

To assess cell cycle variation, cells were trypsinized, washed with PBS, and fixed with 70% cold ethanol at 4°C overnight. Fixed cells were stained with 50 mg/mL propidium iodide in the presence of 1 mg/mL RNase A for 30 min at room temperature. Finally, samples were analyzed by flow cytometry.

### EdU immunofluorescence staining assay

The EdU kit (RiboBio Co. Ltd., China) was used to further validate cell viability. Approximately 5 × 10^5^ cells were plated in 12-well plates with coverslips and were allowed to adhere. Percentage of EdU-positive cells was calculated according to the manufacturer's protocol.

### Microarray analysis

To identify alterations of gene expression profile following PON3 downregulation, microarray experiments and analyses were performed. The cDNA microarrays were constructed as previously described [37]. The threshold used to screen differentially expressed genes is fold change ≥ 1.5 and *p*-value ≤ 0.05. GO (http://geneontology.org/) and KEGG pathway (http://www.genome.jp/kegg/pathway.html) analyses were performed using the standard enrichment computation method and ranked by *p*-values [38].

### Statistical analysis

All statistical analyses were performed using the SPSS version 17.0 and the GraphPad Prism 5.0 software. For qualitative variables, χ^2^ test or Fisher's exact test was used. For continuous variables, Student's *t*-test or the Mann-Whitney test was performed as appropriate. Survival curves were calculated according to the Kaplan-Meier method and were compared by a log-rank test. The Cox's proportional hazards model was used to determine independent factors of survival and recurrence based on variables selected after the univariate analysis. Two-tailed tests were performed to generate *p*-values, *p* < 0.05 was considered statistically significant.

## SUPPLEMENTARY MATERIALS



## Abbreviations

HCC: hepatocellular carcinoma
PON1/2/3: paraoxonase1/2/3
RFS: recurrence-free survival
OS: overall survival
TNM: tumor node metastasis
COX1/2: cytochrome c oxidase subunit 1/2
ROS: reactive oxygen species
AFP: alpha fetoprotein
MKI67: marker of proliferation Ki-67
PAX6: paired box 6
IL18: interleukin 18
IGFBP2: insulin like growth factor binding protein 2
MMP7: matrix metallopeptidase 7
NUPR1: nuclear protein transcriptional regulator 1
CENPF: centromere protein F.
