# Supplementary material for: Paraoxonase 3 inhibits cell proliferation and serves as a prognostic predictor in hepatocellular carcinoma
Source: Oncotarget. 2016 Sep 20;7(43):70045–57. doi: 10.18632/oncotarget.12145 (PMC5342533; doi:10.18632/oncotarget.12145)
Supplement: Supplementary file 1 [file oncotarget-07-70045-s001.pdf]

Paraoxonase 3 inhibits cell proliferation and serves as a prognostic predictor in hepatocellular carcinoma

Supplementary Materials

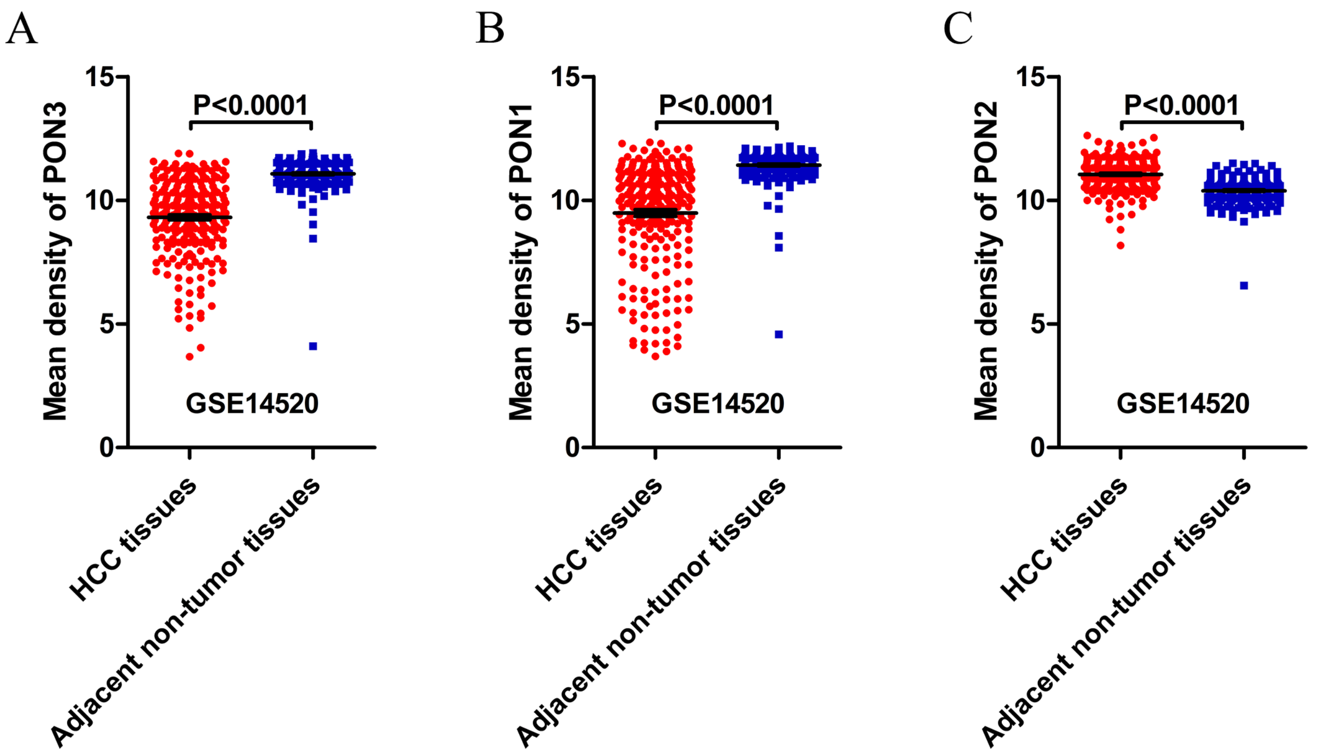

Supplementary Figure S1: GEO DataSets (GSE14520) analyses of PON genes expression in HCC. Statistical significance was determined by student's *t*-tests. Data are shown as mean ± SD.

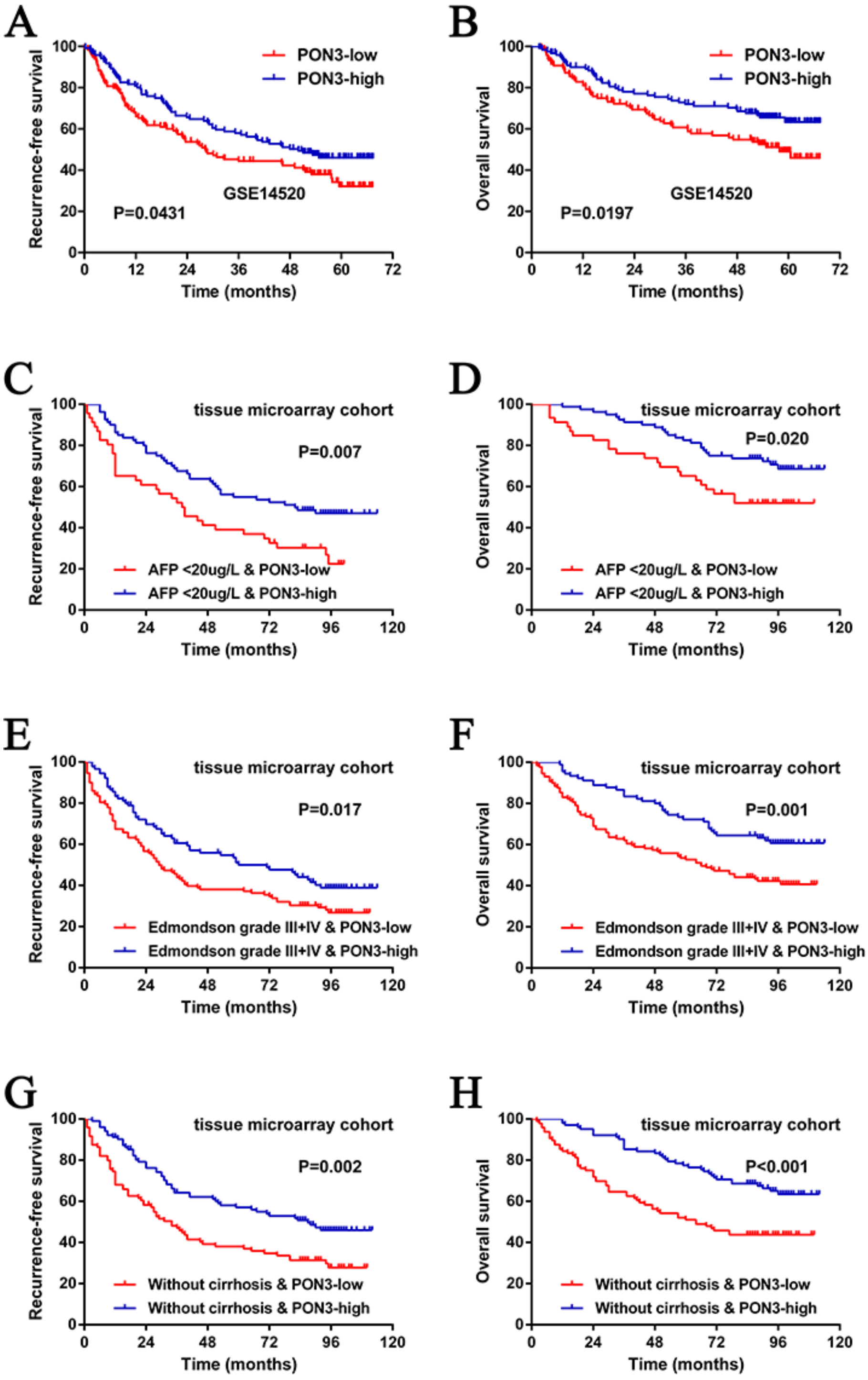

Supplementary Figure S2: (**A** and **B**) The subgroup with low PON3 mRNA level had significantly shorter RFS and OS than the subgroup with high PON3 mRNA level in an online microarray data (GSE14520). The prognostic value was also observed in patients with serum AFP < 20 µg/L (**C** and **D**), poor differentiation (**E** and **F**), and cirrhosis absence (**G** and **H**). Statistical significance was assessed by two-sided log-rank tests.

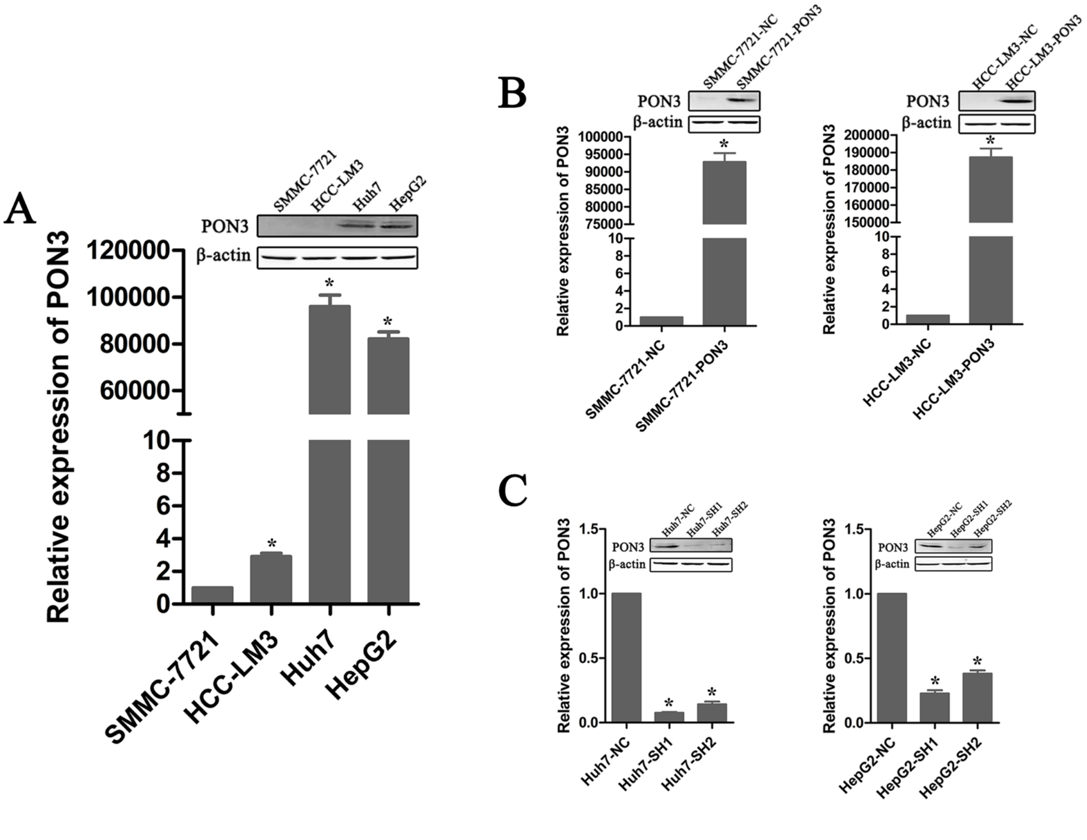

Supplementary Figure S3: (**A**) PON3 expression level in HCC cell lines. (**B** and **C**) The efficiency of PON3 overexpression or knockdown in stable transfected cells. Data are shown as mean ± SD. Significance ($p < 0.05$) was determined from three independent experiments and assessed by student's *t*-test.

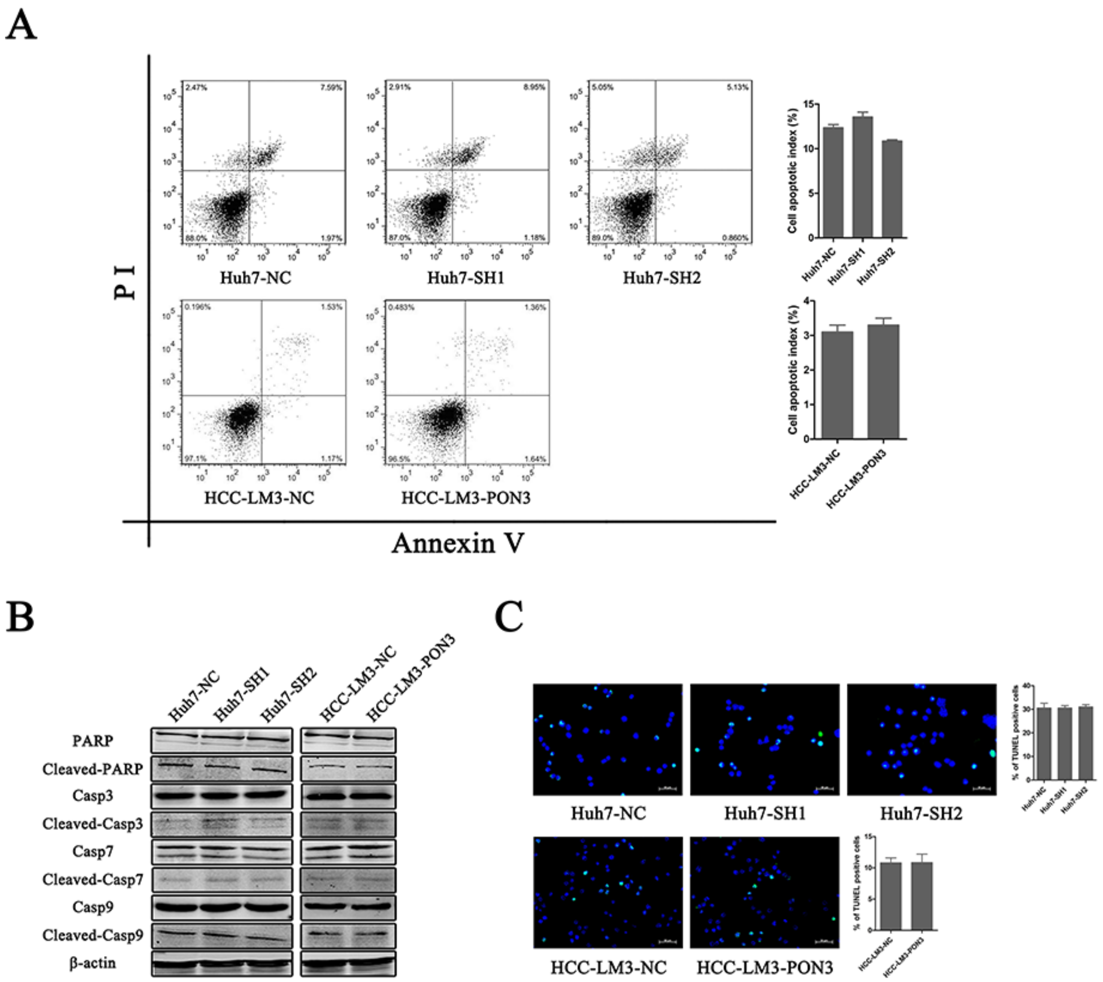

Supplementary Figure S4: Anti-apoptotic function of PON3 was not observed in HCC cells. (**A**) A flow cytometry analysis of apoptosis using annexin V-FITC and PI staining in PON3 overexpression and knockdown cells. (**B**) Western-blot analysis of apoptosis-related proteins PARP, caspase-3, -7, -9 and their respective cleavage fragments in PON3 overexpression and knockdown cells. β-actin was used as an internal control. (**C**) Terminal deoxynucleotidyl transferase dUTP nick end labeling (TUNEL) staining of PON3 overexpression and knockdown cells. Nuclei were detected with Hoechst 33342 in blue and TUNEL-positive cells were in green. Magnification, 200×; scale bars = 50 µm. Cells were pretreated with apoptosis-inducers A (Apopida) and B (Apobid) (1:1,000) for 6 h for apoptosis analyses. The experiments were performed in triplicate and data are expressed as the mean ± SD. Significance was assessed by student's *t*-test.

Supplementary Table S1: Univariate analysis of the post-hepatectomy outcomes of HCC patients in tissue microarray cohort

Variable	Recurrence-Free Survival (%)▲	*P*-value	Overall Survival (%)▲	*P*-value
Age (year), ≥ 50: < 50	36.232:36.486	0.589	53.623:52.027	0.764
Gender, Male: Female	35.827:40.625	0.533	50.394:71.875	0.045★
Hepatocirrhosis, Present: Absent	27.273:40.404	0.018★	48.863:54.545	0.437
Edmondson grade, III + IV: I + II	35.616:38.806	0.234	49.772:62.687	0.036★
Tumor size (cm), ≥ 5: < 5	36.842:36.126	0.154	42.105:58.115	0.001★
Capsule, Absent: Present	41.975:34.146	0.476	49.383:54.146	0.216
Microvascular invasion, Present: Absent	35.000:36.466	0.443	40.000:53.759	0.051
Satellite lesions, Present: Absent	22.727:37.500	0.020★	50.000:53.030	0.598
TNM stage, II-IV: I	26.230:39.111	0.001★	36.066:57.333	0.001★
HBeAg, Positive: Negative	35.922:36.612	0.708	49.515:54.645	0.298
Serum AFP (ug/L), ≥ 20: < 20	33.750:39.683	0.031★	44.375:63.492	0.000★
PON3 expression, Low: High	32.168:40.559	0.007★	44.056:61.538	0.000★

▲The time follow-up ended is used to calculate the Recurrence-Free Survival and Overall Survival
★$p < 0.05$ by Long-rank test.

Supplementary Table S2: The list of primers sequences used in study

Gene symbol	Sequence
**PON3**	Forward: GAGAAGTGGAGCCAGTAGA
	Reverse: CAGGAGGGAGTTGGTAAA
**MKI67**	Forward: AATTGAACCTGCGGAAGA
	Reverse: CTCTCCTCTGCCACCTT
**PAX6**	Forward: GAAGCAAGAATACAGGTATGG
	Reverse: TTGTGGAATTGGTTGGTAGA
**IL18**	Forward: TGAATCCTCCTGATAACATC
	Reverse: TAGTCTTCGTTTTGAACAGT
**IGFBP2**	Forward: CCAAGAAGCTGCGACCAC
	Reverse: GGGATGTGCAGGGAGTAGAG
**MMP7**	Forward: ACGGATGGTAGCAGTCTA
	Reverse: ATGGAGTGGAGGAACAGT
**NUPR1**	Forward: ACAGAGCTGGAGATGAGG
	Reverse: TGCCGTGCGTGTCTATT
**CENPF**	Forward: GCAGAAGACACGGAAGG
	Reverse: GGTTCTCGGAGGATGGT
**β-actin**	Forward: GGGAAATCGTGCGTGACATTAAG
	Reverse: TGTGTTGGCGTACAGGTCTTTG
